# Visual strategies of young soccer players during a passing test – A pilot study

**DOI:** 10.16910/jemr.15.1.3

**Published:** 2022-02-21

**Authors:** Pieter Vansteenkiste, Matthieu Lenoir, Izabela Krejtz, Krzysztof Krejtz

**Affiliations:** Ghent University, Belgium; SWPS University of Social Sciences and Humanities, Warsaw, Poland

**Keywords:** Gaze behavior, motor learning, perceptual learning, Sport, Football

## Abstract

In sports, studies on visual behavior have mostly focused on expert-novice differences during decision making tasks and during aiming tasks. How visual behavior changes during the early stages of skill acquisition however, has hardly been documented. The current study investigated gaze behavior of young soccer players during the execution of a soccer passing task. Gaze behavior of eleven 8- to 10-year-old soccer players was recorded while they performed the Loughborough Soccer Passing Test. Based on their score, participants were then divided into a high performance group (N=5), and a low performance group (N=6). Results showed that the low performance group tended to look more at the ball while they were handling it. These findings suggest that gaze strategies develop alongside technical skills. More insights in the interplay between technical skills and gaze strategies during skill acquisition could lead to improved training methods for young soccer players.

## Introduction

Skilled athletes have consistently been found to outperform less skilled athletes in perceptual-cognitive tests such as pattern recall, anticipation, and decision making (1; 32). Eye tracking experiments have shown that these superior perceptual-cognitive skills of skilled athletes are often supported by different gaze strategies (17). However, how exactly experts’ gaze strategies differ from those of novices seems to vary strongly between experiments. For example, expert performance in sports has been linked to both longer and shorter fixations (13). A longer fixation duration of expert athletes has typically been interpreted as experts extracting more information from each fixation. Shorter fixation durations on the other hand, have been linked to more efficient processing of visual information (as predicted by the theory of long-term working memory; 8). Although these findings seem contradictory, they can be explained by the fact that gaze behavior is highly dependent on environmental and task constraints. Overall, it can be concluded that expert performers adopt a more effective and task-specific gaze strategy. How exactly this gaze strategy is different from that of novices depends on the sport-specific task requirements, but also on the research paradigm, and on the stimulus presentation (18).

Visual information does not only serve for anticipation and decision making, but is also essential to plan and/or guide motor actions. When steering through curves, the ‘tangent point’ has been found to be an important point of fixation to guide steering (14; 27). In ball sports such as tennis and cricket, the eyes have been found to make anticipatory saccades towards the point where the ball will bounce and/or where the ball will be hit (15). And finally, when aiming in basketball or golf, the final fixation towards a specific location just prior to movement initiation (known as the Quiet Eye), has repeatedly been shown to be longer in expert athletes and in successful trials (29; 30). These task-specific gaze strategies have been identified to be more robust in expert performers and have been subject to training studies for novices.

Although there are ample studies describing expert-novice differences in gaze behavior, and literature about how to train perceptual-cognitive skills in sport is growing, little is known on how visual strategies come about during the early stages of skill acquisition. Visual behavior is seemingly expected to develop naturally alongside motor skills (6). Whereas motor development and motor learning has been extensively studied, little is known about how visual behavior changes alongside motor skills. Experiments in juggling have shown that as motor expertise increases, participants develop different gaze strategies characterized by a shift of relying on foveal vision to track the balls, to relying on peripheral vision (11). This shift of visual strategy emerges without explicit instruction, and it is even highly questionable if instructing a novice juggler to adopt an expert’s strategy would be beneficial. Currently, little is known about the interaction between perceptual and motor skills during skill acquisition.

As is the case for other sports, visual and perceptual studies in soccer have primarily focused on anticipation and decision making. Successful decision-makers in football have been found to spend more time fixating the player in possession of the ball (25; 22), and to make more exploratory head movements to acquire information on the position of opponents and teammates (often referred to as scanning; 10; see 18 for a review on scanning in soccer). However, visual behavior during ball handling has been less thoroughly studied.

Recently, Natsuhara et al (19) studied soccer players’ visual search strategies when making a passing decision. Although also a decision making task, in this experiment the decision had to be made while receiving and passing a ball. Both high and low level players predominantly gazed at the ball when it was approaching, but high level players focused more on the free teammates and opponents prior to receiving the ball.

To our knowledge, no research has been carried out which investigated how visual strategies develop during soccer specific skill acquisition. The aim of the current study was therefore to investigate to what extent ball handling skills of young soccer players who are still learning basic ball handling skills can be linked to their gaze behavior.

## Methods

### Participants

Nineteen participants were recruited from a local football academy. All participants were between 8 and 10 years old and had about 2 years of experience in the academy. Written informed consent was obtained from all participants’ parents. After an initial qualitative analysis, the eye tracking data of 8 participants was excluded from further analysis due to low calibration quality, or poor eye movement registration (see data analysis).

### Materials

**Figure 1.**
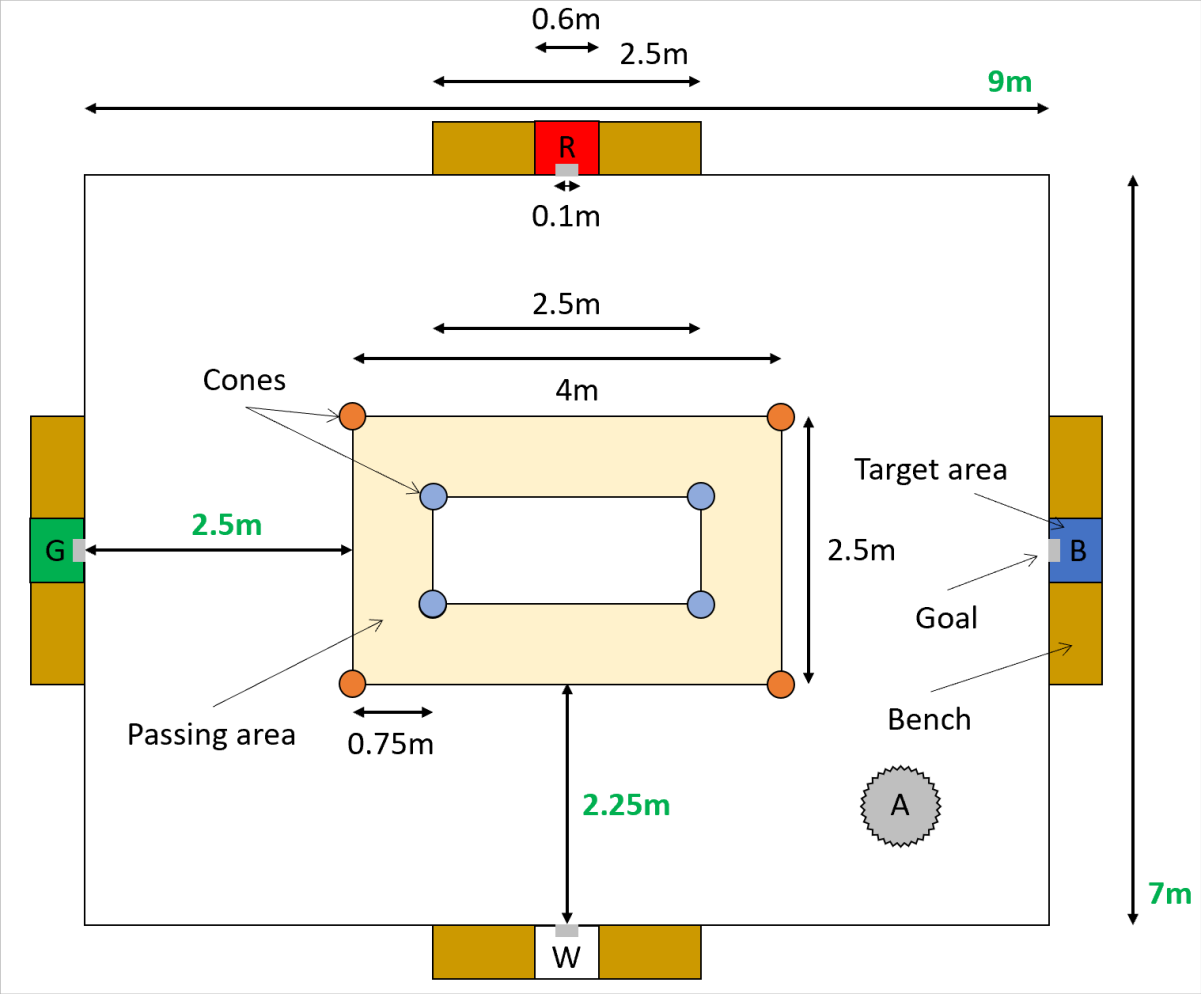
Overview of the set-up. Distance between the center square and the benches were adapted to fit the available space. Dimensions which differ from the original LSPT are in Green. ‘R’, ‘G’, ‘B’ and ‘W’ mark the red, green, blue and white targets, respectively. ‘A’ indicates a support column (adapted from 2).

**Figure 2.**
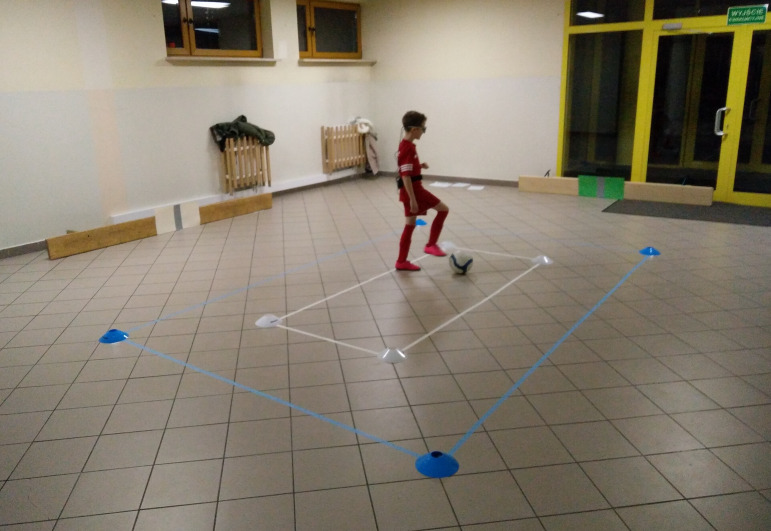
Participant with eye tracker ready to start the LSPT. The white and the green target can be seen on the two benches, the grey strip in the middle of the colored target is the goal. Passing area is between the blue and white lines on the floor. A gaze-overlay video of one of the participants can be watched via https://youtu.be/IL9z9fYCcho.

Participants carried out a Loughborough Soccer Passing Test (LSPT; 16) in the entrance hall of a gymnasium. However, as this hall was smaller than the dimensions of the original LSPT, the layout of the test was adapted to fit the available space (see fig. 1). In the LSPT, participants are required to pass to one of the four goals (10cm wide), located on colored targets (60cm wide) from a marked passing area (see fig. 1 & fig. 2). The color of the next target was called out by the experimenter immediately succeeding each pass. Time to complete the test was measured from the moment the ball entered the passing zone the first time, to the moment the final (16th) target was hit. Further details on the LSPT can be found in Le moal et al. (16).

The Eye Tracking Glasses 2 wireless (ETG2w, SMI, Teltow, DE) were used to record eye movements during the test. The system recorded eye movements at 60hz, while a frontal camera recorded the scenery in front of the participant at 24Hz. The glasses were connected to a smartphone (Samsung Galaxy Note 4) which was carried in a pouch. The system was calibrated using a 3-point calibration, and the tracking accuracy of the system was below 0,5°.

### Procedure

At arrival the participants were asked to put on the eye tracking glasses and performed the calibration procedure. They were then explained how to carry out the LSPT and were informed to carry out the test as quickly as possible while making as few mistakes as possible. One experimenter called out the colors where the participant had to pass the ball, while another experimenter kept time and recorded the penalties. A third experimenter kept other participants and visitors (participants’ parents) away from the test area while a test was in session. Each participant completed one trial of the LSPT. This experiment was approved by the ethical committee of Ghent University (EC UZG 2017/1548).

### Data analysis

The score on the LSPT is calculated by adding penalty points to the trial duration (in seconds). Penalties: +5s for missing the bench/passing to the wrong target, +3s for missing the target area, + 3s for handling the ball, +2s for passing from outside of the passing area, +2s for touching any cone, -1s for hitting the goal. Based on the LSPT score, the participants were categorized into a ‘high performance group’ (score < 80, N = 5), and a ‘low performance group’ (score > 80, N = 6).

Using BeGaze 3.7, the eye movements were superimposed on the 24Hz frontal scene video images and exported as a video file. These video files were then inspected for data quality. A participant was excluded when a) the calibration was too poor to reliably estimate the gaze location, b) gaze was located outside of the video frame too often, and/or c) too much noise was present in the gaze location due to an artifact being erroneously tracked as the pupil. For the remaining 11 participants, a frame-by-frame gaze location analysis (28) was performed in Kinovea (at 24Hz). For each frame, gaze was categorized to one of the following Areas Of Interest (AOI): 1) Target bench 2) Non-target bench, 3) Cones and lines, 4) Ball while handling, 5) Ball while not in possession, 6) Floor, 7) Other (e.g., experimenter or wall), 8) Unknown/out of screen (Gaze location could not be determined). Based on this analysis, dwell time percentages to each of the AOIs was calculated. Dwell time percentages were then compared between the high-performance group and the low performance group using Mann-Whitney U tests.

## Results

The high-performance group had a significantly lower score for the LSPT than the low performance group (76.00 ± 4.30 and 96.83 ± 12.50, respectively; MWU = 0.000; Z = -2.739; p = 0.006; Cohen’s d = -1.53), completing the test both in a shorter time (59.60 ± 5.03s vs. 72.33 ± 11.78s, respectively), and with fewer penalties (16.40 ± 3.51 vs. 24,50 ± 3.78, respectively). The high-performance group tended to look less at the ball while they were in possession than the low performance group (Z = -1.826; p = 0.068), and spent less time looking at ‘other’ regions such as the experimenters (Z = -2.008; p = 0.045). All other differences were non-significant (p > 0.1; see table 1 for details).

**Table 1:** Dwell time percentages to each AOIs

	High performance	Low performance	Mann-Witney U	Z	p	Cohen's d
	(LSPT < 80)	(LSPT > 80)				
Target bench	9.54 ± 6.31	13.54 ± 5.89	8	1.278	0.201	-0.66
Non-target bench	0.96 ± 0.85	0.68 ± 1.08	12	0.831	0.406	0.28
Cone/line	11.05 ± 8.11	11.02 ± 4.56	11.5	0.548	0.584	0.00
**Ball while handling**	**2.97 ± 2.37**	**11.15 ± 7.12**	**4**	**1.826**	**0.068**	- 1.48
Ball while not in possession	20.37 ± 3.04	17.88 ± 7.33	10.5	0.730	0.465	0.43
Floor	42.43 ± 7.72	34.82 ± 7.55	7.5	1.278	0.201	1.00
**Other**	**0.74 ± 0.7**	**2.80 ± 2.21**	**4.5**	**2.008**	**0.045**	-1.20
Unknown	11.95 ± 11.54	8.10 ± 4.78	14.5	0.000	1.000	0.45

## Discussion

Participants who possessed better ball handling and passing skills adopted a different visual strategy than their less skilled peers, characterized by looking less at the ball while handling it, and paying less attention to task irrelevant areas (‘other’). This suggests that also in soccer, gaze strategies develop alongside technical skills. These findings are in line with earlier findings that attention demands decrease with improving skills (4), and have some practical implications for youth soccer trainers.

As learning soccer players become more proficient in a certain task, they move from the cognitive stage into the associative stage of motor learning (7). This comes with a reduced cognitive effort to carry out the same task. Consequently, visual attention can gradually shift away from the ball when handling it, potentially relying more on peripheral vision to track the ball. This allows more experienced soccer players to pay more attention to their surroundings without this affecting their ball handling task (23).

It is not clear however if this shift of visual attention always emerges on the same moment during the skill acquisition. Possibly, an inappropriate distribution of visual attention could cause delays in motor learning. A player with inadequate ball handling skills could be paying too much attention to his/her surroundings, jeopardizing the motor learning process. Vice versa, a skilled player could still be paying too much attention to the ball, jeopardizing the progress in tactical decision making. A trainer should therefore always take into account the technical skills of a player when suggesting where to look at (e.g., ‘keep your eyes on the ball’).

It is also interesting to note that no differences in dwell time percentage were found for looking at the ball while not being in possession. This could indicate that receiving a ball was too demanding for both groups to direct visual attention elsewhere. Possibly, receiving a ball using peripheral instead of foveal vision is only possible for older and/or more skilled players (11). This also underlines that different tasks have different cognitive demands, and therefore require different visual strategies. Feedback on where to look should therefore be tailored to both the individual’s stage of motor learning and the cognitive demands of the task.

The question remains if teaching an ‘expert gaze pattern’ to a young soccer player will benefit his/her motor skills. Gaze training has shown to improve performance in perceptual and basic motor coordination tasks (12; 31), and even in more complex motor tasks such as gymnastics and golf swinging (9; 5). Nevertheless, as mentioned earlier, some gaze patterns might only be feasible with a higher level of motor skill (e.g. receiving a ball using only peripheral vision). It is therefore possible that teaching young soccer players where to look might affect their gaze behavior, but will not improve their motor performance (3; 20). Furthermore, it also needs to be taken into account that the same action could be successfully executed using more than one gaze strategy (26; 24). For example, Futsal players have been found to acquire visual information just prior to ball control, while in contrast, soccer players executing the same task scan the environment when not in possession of the ball (21). The ‘ideal’ gaze strategy for any motor task might therefore also be different depending of individual and/or environmental constraints.

Unfortunately, the current sample only included 11 participants and was performed using a cross-sectional analysis. Following the perceptual-motor development of young soccer players as they develop soccer skills would provide more insights into the interplay between perceptual-cognitive development and motor development. This in turn could lead to more tailored feedback based on the stage of motor learning. The current study purposely tested young soccer players as they are still developing their basic ball handling skills. However, this comes with the disadvantage that the results might not only reflect differences in soccer skills, but also in general cognitive and/or motor development. The development of perceptual motor skills as an adult might be different than as a child.

Testing child participants in a highly dynamic situation is a very challenging environment to collect eye movement data. Not only are the eye tracking glasses designed for adults, the LSPT also required frequent and fast head movements. Despite having fastened the eye tracking glasses with a head strap, in some participants, the glasses still moved too much during the experiment to result in reliable data. Future studies might therefore consider using adapted eye tracking glasses when testing children in a dynamic environment.

Next to the hardware-related challenges, the current study also had to deal with some challenges concerning data analysis. Although in some cases it can be appropriate to use fixation detection algorithms to analyze gaze behavior acquired with a head mounted eye tracker (28), in general, this method is considered unreliable. In a dynamic situation (such as in the current study), eye movements are affected by optokinetic and vestibulo-ocular reflexes, and regularly involve smooth pursuit movements. As fixation detection algorithms deal poorly with these kinds of eye movements, both the duration and location of fixations that are detected using these algorithms are often not reliable. As a result, data of the current study was analyzed using the tedious frame-by-frame method. Future studies should focus on gaze behavior during the execution of one specific task (such gaze just prior to the reception of a pass), rather than analyzing the average gaze behavior over multiple actions. This will keep time-consuming analyses feasible, and will help avoiding to ‘average out’ the results of two (or more) different tasks.

In summary, the current study showed that young soccer players who already possessed better ball handling skills tend to look less at the ball while handling it, and pay less attention to task irrelevant areas. This implies that gaze strategies develop alongside technical skills. During the technical skill acquisition stage, feedback on where to look should be tailored to both the individual’s stage of motor learning and the cognitive demands of the task.

### Ethics and Conflict of Interest

The author(s) declare(s) that the contents of the article are in agreement with the ethics described in http://biblio.unibe.ch/portale/elibrary/BOP/jemr/ethics.html and that there is no conflict of interest regarding the publication of this paper.

### Acknowledgements

We thank Weronika Węgier, Julia Adamus, Dominik Kudła, Monika Kowalska, Joanna Nowakowska, Agnieszka Ozimek, Aleksandra Piotrowska, Katarzyna Żukow – Tapioles for their assistance in data collection. We would also like to thank Mr. Szeliga and Rafał Wielądek, soccer trainers and Szel-Gol Football Club from Ząbki, Poland for the cooperation and the possibility to run research with their team. Finally we also thank Ollie Gibbins for helping with data analysis, and Ignacio Drebert for all the love and support.
